# Adaptive and Energy-Efficient Optimal Control in CPGs Through Tegotae-Based Feedback

**DOI:** 10.3389/frobt.2021.632804

**Published:** 2021-05-26

**Authors:** Riccardo Zamboni, Dai Owaki, Mitsuhiro Hayashibe

**Affiliations:** ^1^ Politecnico di Milano, Milan, Italy; ^2^ Department of Robotics, Graduate School of Engineering, Tohoku University, Sendai, Japan

**Keywords:** central pattern generator, sensory feedback, tegotae approach, efficiency, optimal control, learning, embodiment

## Abstract

To obtain biologically inspired robotic control, the architecture of central pattern generators (CPGs) has been extensively adopted to generate periodic patterns for locomotor control. This is attributed to the interesting properties of nonlinear oscillators. Although sensory feedback in CPGs is not necessary for the generation of patterns, it plays a central role in guaranteeing adaptivity to environmental conditions. Nonetheless, its inclusion significantly modifies the dynamics of the CPG architecture, which often leads to bifurcations. For instance, the force feedback can be exploited to derive information regarding the state of the system. In particular, the *Tegotae* approach can be adopted by coupling proprioceptive information with the state of the oscillation itself in the CPG model. This paper discusses this policy with respect to other types of feedback; it provides higher adaptivity and an optimal energy efficiency for reflex-like actuation. We believe this is the first attempt to analyse the optimal energy efficiency along with the adaptivity of the Tegotae approach.

## 1 Introduction

The ability to efficiently move in complex environments is a key property for animals and their survival. This implies that many aspects of their morphology and central nervous system are shaped by constraints related to their locomotor skills. Animal locomotion is not generated merely from neural systems; instead, it is generated from the close interaction between neural systems, musculoskeletal systems, and the real-world environment (Pfeifer and Bongard, 2006; Pfeifer et al., 2007). Thus, it is essential to elucidate the locomotion generation mechanism by analysing the interaction dynamics among these three systems and by analysing the neural systems themselves. Understanding these mechanisms is expected to result in contributions to biology and robotics by facilitating the design of durable and resilient robots that are energy-efficient.

Central pattern generators (CPGs) are neural circuits that are found in invertebrate (Pearson and Iles, 1973; Bässler and Wegner, 1983; Bässler, 1986) and vertebrate animals (Shik et al., 1966; Grillner, 1975; Grillner, 1985). CPGs can produce rhythmic patterns of neural activity without receiving any rhythmic inputs. The term *central* indicates that the sensory feedback from the *peripheral* nervous system is not needed for generating the rhythms (Marder and Bucher, 2001; Ijspeert, 2008). Biological CPGs underlie many fundamental rhythmic activities such as chewing, breathing, and digesting. In addition, they also serve as the fundamental building blocks for locomotor neural circuits. From the perspective of control, they have several interesting characteristics such as a distributed control, the ability to deal with redundancies, the presence of fast control loops, and the ability to modulate the locomotion by using simple control signals. Owing to these properties, CPGs are considered to be transferred mathematical models. In addition, CPGs serve as the building blocks of robotic locomotion controllers and are being increasingly used in the robotics community (Ijspeert, 2008). To enable biologically inspired robotic control, the architecture of CPGs has been extensively adopted to generate periodic patterns for locomotion control owing to the properties of nonlinear oscillators (Kimura et al., 1999; Fukuoka et al., 2003; Tsujita et al., 2003; Aoi and Tsuchiya, 2005; Buchli et al., 2006; Kimura et al., 2007; Righetti and Ijspeert, 2008; Wang et al., 2011).

Although sensory feedback in CPGs is not necessary for generating rhythmic patterns, it plays a central role in guaranteeing adaptivity to the environmental conditions (Ijspeert, 2008).

Sensory feedback in CPGs for animal locomotion was first studied in the pioneering work on bipedal walking conducted by Taga et al. (1991), Taga (1994), Taga (1995). In these studies, sensory information from the environment was fed back into the nervous system model to generate a walking pattern from the interaction among the nervous system model, musculoskeletal model, and environment (“Global Entrainment”). Kimura et al. (1999); Fukuoka et al. (2003) proposed a model by integrating CPG and reflex mechanisms to realise uneven terrain quadruped walking. Aoi and Tsuchiya (2005), Aoi and Tsuchiya (2006) focused on “phase resetting” (Schomburg et al., 1998), a feedback mechanism found in animals, to include gait stabilisation in CPG-based control models. Aoi’s group also applied the phase resetting feedback in CPGs to human-like musculoskeletal models of bipedal walking (Aoi et al., 2010), quadrupedal gait transitions (Aoi et al., 2011; Aoi et al., 2013), and a hexapod walking model (Ambe et al., 2018). Steingrube et al. (2010); Manoonpong et al. (2010) proposed a modular neural control with bio-inspired CPG-based network and sensory feedback, demonstrating environmental adaptability, such as walking on uneven terrain and avoiding unknown obstacles, and then extended the models by introducing forward models (Manoonpong et al., 2013; Dasgupta et al., 2015), visual feedback (Goldschmidt et al., 2014; Grinke et al., 2015), muscle models (Xiong et al., 2014; Xiong et al., 2015), and so on. Buchli et al. (2006); Nachstedt et al. (2017) proposed an adaptive frequency oscillator that could learn motion frequency adaptively and verified the generation of gait according to body characteristics. Furthermore, an interlimb coordination model that employed load information as sensory information and generated adaptive and diverse quadruped walking patterns was proposed (Maufroy et al., 2010; Fukuoka et al., 2015; Owaki and Ishiguro, 2017a). Sensory feedback inclusion significantly modifies the dynamics of the CPG’s architecture, which often leads to bifurcations and other dynamic phenomena (Aoi et al., 2011; Wang et al., 2011; Aoi et al., 2013).

To establish a systematic design principle of the sensory feedback in the CPGs to achieve biologically inspired robotic locomotion, a novel concept called “Tegotae” is proposed. Tegotae is a Japanese concept that describes the extent to which a perceived reaction matches the intended motor command. The potential of the Tegotae approach in reproducing animals’ locomotion and understanding the underlying mechanism has been previously demonstrated based on synthetic approaches. The Tegotae approach was first used by Owaki et al. (2017) to develop a minimal model for interlimb coordination on hexapod robot locomotion with CPG-based control. Kano et al. (2018) demonstrated gait transition between the concertina and scaffold-based locomotion in a snake model simulation with reflex-based control. Kano et al. (2019) proposed the detailed design of the Tegotae function, particularly for motor commands, using the genetic algorithm (GA) to simulate a simple 1-D earthworm model with CPG-based control. Owaki et al. (2021) demonstrated adaptive walking control on a biped model with CPG and reflex-based controllers.

The main contribution of this study is the construction of a specific proprioceptive feedback law through the so-called Tegotae approach (Owaki et al., 2017). Together with a specific control policy, i.e. reflex-like actuation, it exploits it fruitfully based on the concept of embodied intelligence (Pfeifer and Bongard, 2006; Pfeifer et al., 2007). Then, the feedback is applied to certain mechanical systems, i.e. hopping systems; is first considered for the simplest case of one leg, and is then extended to two legs. In such circumstances, the sensory feedback plays an important role in shaping the rhythmic patterns and ensuring coordination between the CPGs and body movements. This study demonstrates the adaptation processes as well as the acquirement of the different gait. In addition, it compares the analytical solution for the single-leg case with an optimal controller solution that is based on direct methods such as the multiple shooting methods (Bock and Plitt, 1984; Diehl et al., 2005; Fagiano, 2019). This confirms the intuitions for the energy efficiency of the control policy. Finally, we extensively analyse the approach in relation with the considerations for learning and energy efficiency (Hayashibe and Shimoda, 2014).

The following section presents the materials and methods used in this study. First, we briefly describe the Tegotae approach. Second, we present the mathematical model for the Tegotae-based control. Third, we discuss the Tegotae approach based on the learning framework by comparing it with *tacit learning* as described in Hayashibe and Shimoda (2014). Then, we present the simulation results to validate the Tegotae controller and then evaluate the energy efficiency. Finally, in Section 5, we discuss the results and future work.

## 2 Methods

### 2.1 Tegotae Control

#### 2.1.1 Theory

The inclusion of feedback in the architecture of the CPG is a natural extension of these structures. However, any modification to the canonical form leads to a modification in the main dynamics, which may affect the effectiveness. This is achieved by considering a particular family of feedback functions in terms of the local effect of this inclusion on the dynamics of a neural oscillator. The approach to define these feedback functions is called the Tegotae approach, as described in Owaki et al. (2017). Tegotae is a novel concept that describes the extent to which a perceived reaction matches an expectation, or intention, of a controller. Tegotae stems not only from the reaction that is received from the environment, but also from the consistency between the perceived reaction and the intention or expectation of the controller, i.e. what the controller intends to do. In the case of matching, it is said that either “good” or “bad” Tegotae is obtained. In this manner, a cognitive meaning is added to the control framework, in which it denotes some actions as “positive” and others as “negative”. The objective is to maximise the Tegotae function. In this section, the Tegotae formalism is introduced. For the initial step of the investigation, Tegotae is quantified in the simplest mathematical form, i.e. a function that is based on the separation of the variables as follows.

T(u,e)=C(u)S(e) (1)

Hereafter, the function *T* is referred to as the Tegotae function (T-function), which is a function that quantitatively measures the Tegotae. In Eq. (1), *u* represents a control variable and *e* represents the sensory information obtained from multiple sensors that are embedded in the body. The T-function is expressed as the product between *C*(*u*) and *S*(*e*). The former expresses the intention of the controller, while the latter denotes the reaction obtained from the environment. *T* is designed such that it becomes more positive when an enhanced Tegotae is detected. Therefore, for a given T-function, the local sensory feedback *f* is designed in such a way that the control system modulates *u* to increase the amount of Tegotae received. Thus, with regard to the continuous-time systems, *f* is expressed simply as a mono-dimensional gradient system of the T-function *T* with respect to the control variable *u*, as follows.

$$f=\frac{\partial T\left(u,e\right)}{\partial u}$$ (2)

With this formulation, it is possible to systematically design the decentralised controllers by only designing the T-functions that are required. When considering the CPGs’ framework, the *i*-th controller can be first defined as a generic Kuramoto oscillator (Kuramoto (1984)) of phase *ϕ*_*i*_ without the coupling terms but with a specific external field *f*_*i*_ that consists of the local sensory feedback.

$${\dot{\phi }}_{i}=\omega_{i}+f_{i}\left(\phi_{i},e\right)$$ (3)

As a result, this equation leads to the following expression.

$$f_{i}\left(\phi_{i},e\right)=\frac{\partial T_{i}\left(\phi_{i},e\right)}{\partial \phi_{i}}$$ (4)

In Owaki et al. (2017), the T-function was expected to reproduce the hexapedal inter-limb coordination that was observed in insect locomotion by using the Kuramoto oscillators. For this reason, it was generally defined in the first case as follows.

$$T_{i}\left(\phi_{i},N_{i}\right)=\left(-\text{sin}\phi_{i}\right)N_{i}^{V},$$ (5)

where the sensory information *e* consists of the vertical ground reaction forces *N*_*i*_^*v*^ that are acting on each leg. In the basic control of the hexapod robot in Owaki et al. (2017), the leg was controlled to be in the swing phase for *ϕ*_*i*_ = 0 to *π* and the stance phase for *ϕ*_*i*_ = *π* to 2*π* based on the function *C* (*ϕ*_*i*_) = −sin*ϕ*_*i*_ In this formulation, *T*_*i*_ quantifies the Tegotae on the basis of the information that is only locally available at the corresponding leg. When the local controller intends to be in the stance phase (−sin*ϕ*_*i*_ > 0) and receives a ground reaction force (*N*_*i*_^*v*^ > 0), *T*_*i*_ evaluates the situation as “good” Tegotae, and vice versa. As stated above, the reaction in Eq. 1 is generic, and other types of reactions may be taken into account. In our study, the force passing through the body was taken into account, i.e. an elastic force. This definition is inspired by the Golgi tendon organ (Moore (1984)), which is a proprioceptive sensory receptor organ that senses changes in the muscle tension. The T-function is then defined for a generic *i*-th phase oscillator and the feedback signal is expressed as follows.

$$T_{i}\left(\phi_{i},F\right)≜\left(-\sigma \sin\phi_{i}\right)F$$ (6)

$$f_{i}\left(\phi_{i},F\right)=\frac{\partial }{\partial \phi_{i}}T\left(\phi_{i},F\right)=-\sigma \cos\phi_{i}F$$ (7)

where *σ* denotes a proportionality factor and *F* represents the force passing through the body. By the nature of Eq. (6), it follows that this sensory feedback will be absent when there is no contact with the ground.

#### 2.1.2 Tegotae Control Policy: Preliminary Design and Extensions for Reflex-like Actuation

In majority of the CPGs’ controllers, the actuator is driven by a proportional-integral-derivative (PID) control scheme, which compares the actual state of the physical system with the reference signal that was originated by the CPGs’ network (Ijspeert, 2008). One of our main contributions is to attempt to maintain the model-free control approach while taking into account some of the most recent considerations for the above embodied intelligence (Pfeifer and Bongard, 2006; Pfeifer et al., 2007) and control by using neural-like dynamic systems and reflex-like motor control. Buchli et al. (2006) demonstrates the manner in which the neuro-mechanical coupling provided by the feedback forces the secondary dynamics in the phase oscillator; our goal is to analyse and possibly exploit this effect. This study aims to use a critical point for the feedback dynamics, which is a minimum, or a specific section of it, to control the system. This section briefly describes the evolution of the Tegotae control policy towards its current form. In the former control policy law established by Owaki et al. (2017), a constant actuation force with the value *A* was used, and actuation was observed when the phase of the oscillator *ϕ* was within a certain interval containing the selected critical point of the dynamics *ϕ*_0_.

$$\phi_{i}\in \left(\phi_{0}-\text{Δ}/2,\phi_{0}+\text{Δ}/2\right)\Rightarrow F_{a}\left(\phi_{i},\cdot \right)=A$$ (8)

This implies that the force *F*_*a*_ = *A* is applied when the phase *ϕ*_*i*_ ranges from *ϕ*_0_ − Δ/2 to *ϕ*_0_ + Δ/2. It is apparent that a critical factor of this preliminary policy is the on-line adaptation of the values of *ϕ*_0_ and Δ according to the evolution of the dynamics from the transient to the steady state (assuming it is reached), which is non-trivial. In the first instance, these values are considered to be a posteriori once the specific dynamic of the oscillator has been studied and maintained constantly throughout the entire simulation. The results obtained with this simple control policy are analysed in the monoped case study, which demonstrates how even this simple policy can guarantee good performance. Clearly, this policy can be made smoother by substituting the square wave with other types of functions such as bell-shaped trends.

$$F_{a}\left(\phi_{i},\cdot \right)=\frac{A}{\sqrt{2\pi \text{Δ}}}e^{-\frac{1}{2}\frac{\left(\phi_{i}-\phi_{0}\right)}{\sqrt{\text{Δ}}}}$$ (9)

Although this leads to an easier actuation and solves the numerical issues that are introduced by the switching controller, this control policy does not simplify the method of selection of the specific values of *ϕ*_0_ and Δ. In contrast, the entire negative section that is centered around the minimum of the Tegotae feedback can correspond to a critical phase of the entire dynamics. The following expression can be considered.

$$f_{i}\left(\phi_{i},F\right)=\frac{\partial }{\partial \phi_{i}}T\left(\phi_{i},F\right)\leq 0$$ (10)

This specifically indicates that the Tegotae is decreasing. By definition, the aim is to maximise it. It is clear how this area is the designated area to inject a certain force. In particular, this force is required to lead to the maximisation of the Tegotae, which is dependent on the case study. In this study, a positive force leading to a jump satisfies the requirements. Thus, following Eq. (6), the final mathematical form for the reflex-like actuation that is newly proposed in this study is defined as follows.

$$F_{a}\left(\phi_{i},F\right)=-\text{min}\left(0,f_{i}\right)=-\text{min}\left(0,-\sigma \text{cos}\phi_{i}F\right).$$ (11)

**FIGURE 1:**
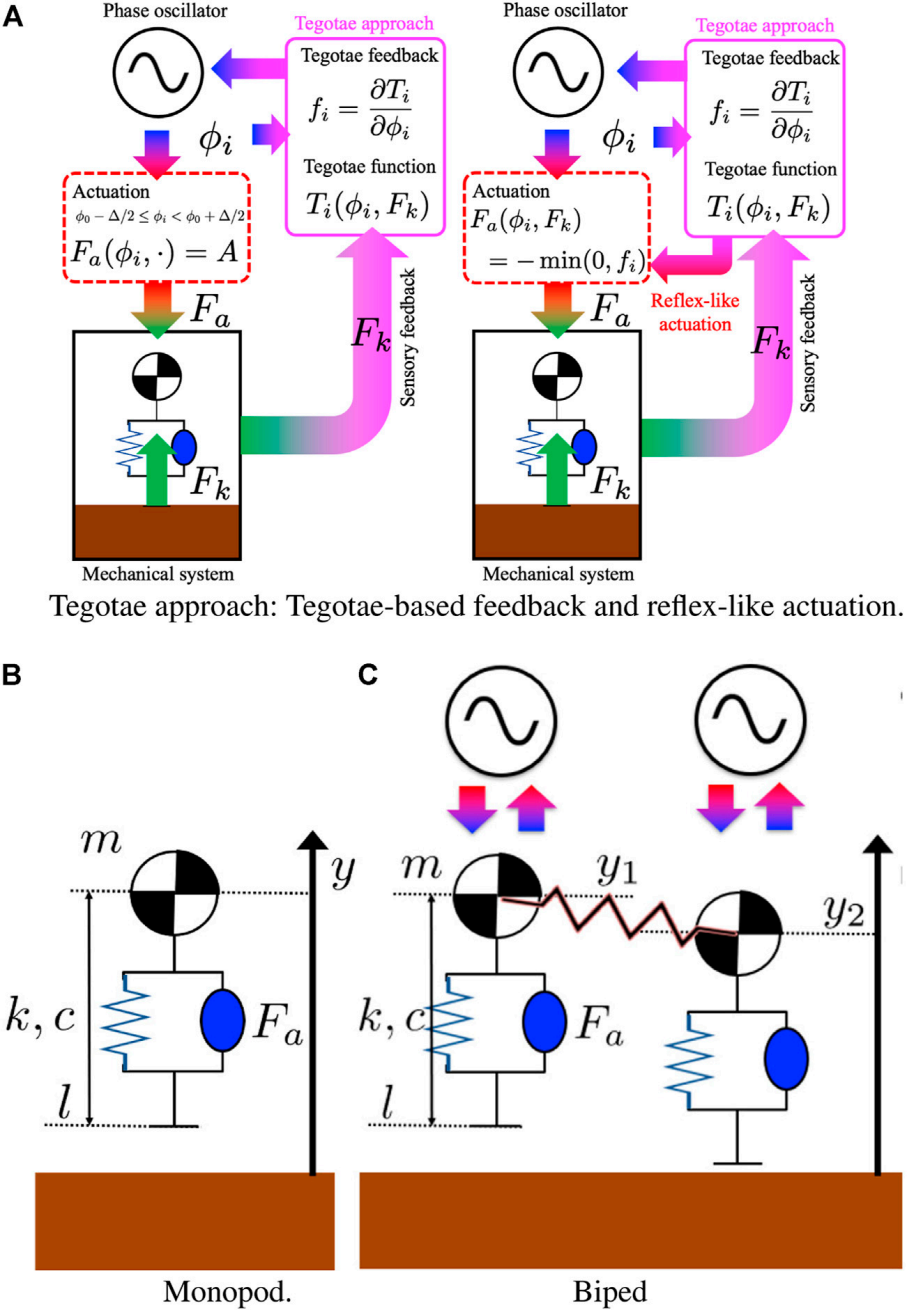
Tegotae approach: **(A)** The reflex-like actuation is designed to be opposite in sign to that of the Tegotae feedback and disappear once the feedback is positive, indicating an increasing Tegotae in Eq. 11. **(B,C)** Neuro-mechanical structure of the mono-dimensional hoppers. **(B)** Monopod: a mass is connected to a mass-less spring and a damper system. A linear actuator is parallel to the spring and damper and it determines the vertical thrust. The Kuramoto model for the phase oscillators was used as a model for the CPGs’ oscillator. **(C)** Biped: Two vertical hoppers are connected with a mechanical spring. Each hopper is controlled by using a decoupled Kuramoto oscillator with Tegotae feedback.

The reflex-like actuation is designed to be opposite in sign to the Tegotae feedback and disappear once the feedback is positive, indicating an increasing Tegotae (Figure 1A). Thus, the negative sign can be attributed to fact that the force actuated in the feedback should be in a direction opposite to that of the force used as the feedback itself. This clearly reintroduces the numerical issues of the switching controller. However, it directly links the actuation and Tegotae feedback in a more biologically inspired reflex-like manner. It also assures an online adaptation to the variation of the dynamics since the Tegotae feedback corresponds to this variation itself, as shown in Figure 1A.

### 2.2 Mechanical Model

#### 2.2.1 Monopod Model

First, a one-dimensional (1-D) hopping system was considered, which is characterised by a mass connected to a mass-less spring and a damper system (Figure 1B). A linear actuator is parallel to the spring and damper and determines the vertical thrust. The Kuramoto model (Kuramoto, 1984) for the phase oscillators was used as a model for the CPGs’ oscillator, simplifying the analysis of the effects of the feedback. The integration of the ordinary differential equations (ODEs) was performed using MATLAB, which automatically stopped the integration when switching was detected. The initial step of the integration was set to 1*e* ^−3^, which is equal to the maximum step of the integration. The evolution of a single phase of the oscillator *ϕ* and the vertical height of the mass *y* is described by an ODE as follows.

$$\dot{\phi }=\omega +f\left(\phi ,F\right),$$ (12)

$$\ddot{y}=\frac{1}{m}\left\{F_{c}\left(\dot{y}\right)+F_{k}\left(y\right)-mg+F_{a}\left(\phi ,F\right)\right\},$$ (13)

$$F_{c}\left(\dot{y}\right)=-c\dot{y},$$ (14)

$$F_{k}\left(y\right)=k\left(l_{0}-y\right),$$ (15)

where *f*(*ϕ*,*F*) is the sensory feedback in the CPG oscillator, while *F*_*k*_(*y*), $F_{c}\left(\dot{y}\right)$, and *F*_*a*_(*ϕ*, ⋅) represent the spring, damper, and actuator force, respectively. These three components are absent during the flight phase, assuming that there no forces that act from the environment.

As previously described, according to Owaki et al. (2017), the Tegotae sensory feedback *f*(*ϕ*,*F*) is defined directly by the Tegotae function *T*(*ϕ*,*F*), where we selected *F* = *F*_*k*_(*y*).

$$T\left(\phi ,F_{k}\right)≜\left(-\sigma \sin\phi \right)F_{k}$$ (16)

$$f\left(\phi ,F_{k}\right)=-\frac{\partial }{\partial \phi }T\left(\phi ,F_{k}\right)=-\sigma \cos\phi F_{k}$$ (17)

with *σ* being a proportionality factor. From Eq. (11), *F*_*a*_ is described as follows:

$$F_{a}\left(\phi ,F\right)=-\text{min}\left(0,f\right)=-\text{min}\left(0,-\sigma \cos\phi F_{k}\right)$$ (18)

Here, as a first step in the evaluation, we used the force passing through the spring *F*_*k*_. An advantage of the Tegotae-based approach is that it can use different forces as sensory feedback. Further extensions may be a combination of many different forces. The novelty of this study lies in the reflex-like actuation equation and the validation of energetic optimality.

#### 2.2.2 Biped Model

The effects of the Tegotae approach on a more complex mechanical and oscillatory system were also studied to prove its effectiveness and ability to sustain different patterns, which were also described by Owaki et al. (2017). The mechanical system was extended to a 1-D bipedal hopping robot as illustrated in Figure 1C. The system corresponds to a slight modification of the previous case.

$${\dot{\phi }}_{1}=\omega_{1}-\sigma F_{k1}\text{cos}\phi_{1}+\epsilon_{12}\text{sin}\left(\phi_{1}-\phi_{2}\right)$$ (19)

$${\dot{\phi }}_{2}=\omega_{2}-\sigma F_{k2}\text{cos}\phi_{2}+\epsilon_{21}\text{sin}\left(\phi_{2}-\phi_{1}\right)$$ (20)

$${\ddot{y}}_{1}=\frac{1}{m_{1}}\left\{F_{c1}\left({\dot{y}}_{1}\right)+F_{k1}\left(y_{1}\right)-m_{1}g+F_{a1}\left(\phi_{1},F_{k1}\right)+F_{k12}\right\}$$ (21)

$${\ddot{y}}_{2}=\frac{1}{m_{2}}\left\{F_{c2}\left({\dot{y}}_{2}\right)+F_{k2}\left(y_{2}\right)-m_{2}g+F_{a2}\left(\phi_{2},F_{k2}\right)+F_{k21}\right\}$$ (22)

In Eqs (19, 20), the Tegotae feedback is already taken into account, while the last term on the right-hand-side represents the weak-coupling between the phase oscillators (Kuramoto, 2003). In Eqs (21, 22) the components are the same as those that are defined in Eq. (12), which is from a simple additional elastic force that is introduced by the connecting spring *F*_*kij*_ = *k*_*c*_(*y*_*j*_−*y*_*i*_). In contrast, the control policy was left unchanged with respect to the monopod case Eq. (18).

## 3 Tegotae in the Learning Framework

The Tegotae approach has certain interesting similarities with other learning frameworks, which motivates some of the intuitions for its energy efficiency. The adaptivity in the learning processes is typically defined for the parameters/weights of the controller/learning agent. In the Tegotae framework, although a further adaptation of the feedback coefficients *σ* may be included, the main adaptation is induced by modifying the dynamics of the oscillators. This factor is taken into account in the comparison, since the eventual adaptation of the parameters is straightforward.

First, it is interesting to note how the Tegotae approach shares some similarities with the *tacit learning*, which is a learning framework that was introduced in Berenz et al. (2014); Berenz et al. (2015). In tacit learning, the control law consists of an extension for the PD controller with a tacit learner block with the time frame (Lt). By using the scalar case for simplicity, the following expression can be obtained.

$$\begin{array}{l} u=kx_{c}^{T}+q\\ q=\int f\left(e\right)dt\;\left(\text{Lt}\right) \end{array}$$ (23)

where *u*, *x*_*c*_, *k*, and *e* are respectively the control, the state variable that is expressed in the control space, the proportional and derivative gain, and any type of quantity that needs to be minimised. The learning process is obtained in the (Lt) block by accumulating the integral over the time of the quantity that needs to be minimised. On this basis, we neglect the proportional and derivative terms in this study.

$$\begin{array}{l} u=q\\ q=\int f\left(e\right)dt\;\left(\text{Lt}\right) \end{array}$$ (24)

The function *f*(*e*) is recommended to have the form *f*(*e*) = *p*(*ξ*)*a*(*e*)^*T*^. In the one-dimensional case, *a*(*e*) can be a simple linear transformation *a*(*e*) = *ae* and *p*(*ξ*) is a periodic function of *ξ*. Both of these additional terms are selected to guarantee the following.

$$\begin{array}{l} \frac{p\left(\xi \right)\;x_{e}}{\left|\left|p\left(\xi \right)\right|\right|\;\left|\left|x_{e}\right|\right|}=1\;\text{if}\;\alpha =0\\ \frac{p\left(\xi \right)\;x_{e}}{\left|\left|p\left(\xi \right)\right|\right|\;\left|\left|x_{e}\right|\right|}=-1\;\text{if}\;\alpha =\pi \end{array}$$ (25)

In Eq. (25), *x*_*e*_ represents the state variable that is expressed in the task space, in which the error *e* is minimized. In contrast, *α* is generically defined as the angle between $\dot{e}$ and *D*(*e*); the latter is the direction toward which *e* is minimized. In the one-dimensional case, α=0∧π. This formulation guarantees that min (*f*(*e*)) = min(*e*). Now, let us consider the Tegotae framework. The objective is to construct feedback and not a feedforward controller. To do this, let us consider the factor that needs to be minimized that corresponds to *e* = −*F*_*k*_, the virtual variable *ξ* to the physical variable *ϕ*, and the error function *a*(*e*) = *σ*e. By neglecting the constant terms due to the integration, the feedback over the oscillator results in the following expressions.

$$\begin{array}{l} u=q\\ e=-F_{k}\\ p\left(\phi \right)=-\text{sin}\left(\phi \right)\\ q=\int f\left(e\right)d\phi =\int \sigma \text{sin}\left(\phi \right)F_{k}d\phi \\ =-\sigma \text{cos}\left(\phi \left(t\right)\right)F_{k}\;\left(\text{L}\phi \right) \end{array}$$ (26)

In the Tegotae framework, *x*_*e*_ = Δ*l* represents the elongation speed of the spring length. This variable points towards the direction of the minimisation of the value of *e* = −*F*_*k*_. Thus, the following expression is obtained.

$$\begin{array}{l} \frac{-\text{sin}\left(\phi \right)\;x_{e}}{\left|\left|\text{sin}\left(\phi \right)\right|\right|\;\left|\left|x_{e}\right|\right|}=1\;\text{if}\;\alpha =0\\ \frac{-\text{sin}\left(\phi \right)\;x_{e}}{\left|\left|\text{sin}\left(\phi \right)\right|\right|\;\left|\left|x_{e}\right|\right|}=-1\;\text{if}\;\alpha =\pi \end{array}$$ (27)

This shows how the Tegotae approach is *de facto* obtaining a tacit learning feedback (Lt) as previously described. Nevertheless, this is achieved by accumulating the quantity that needs to be minimised for the integral of the state space variable that is directly from (L*ϕ*). The integration over the state space frame *ϕ* is coherent with the CPGs’ framework. The role of the oscillators is to provide a different time frame to the dynamics, which is reproduced by the linear transformation *ϕ* = *ωt*. Thus, in the CPGs’ framework, the integration/derivation over the state variable of the oscillator *ϕ* is conceptually equivalent to the integration over the time. Interestingly, it has been demonstrated in Hayashibe and Shimoda (2014) that this controller can guarantee energy efficiency during the task realisation in case the quantity that needs to be minimised is the actuation torque.

## 4 Results

### 4.1 Case1: Monoped

#### 4.1.1 Adaptation Transient and Energy Efficiency

**FIGURE 2:**
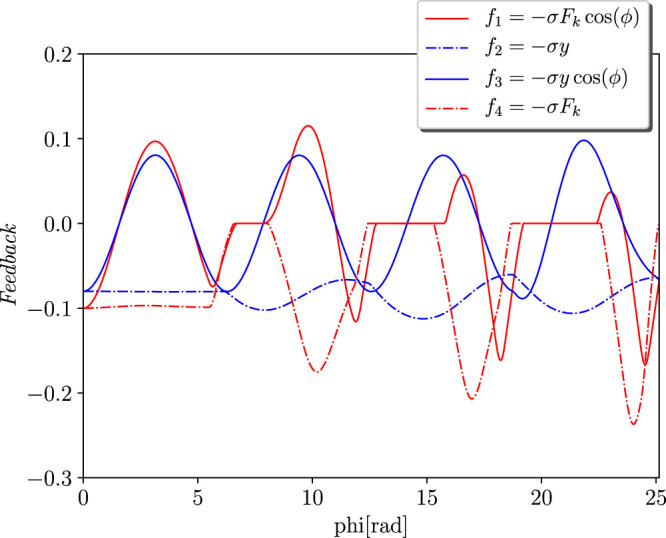
Feedback dynamics over the phase *ϕ*. The different lines represent four different instances for the sensory feedback dynamics. *f*_1_: Tegotae feedback, *f*_2_: height feedback, *f*_3_: feedback in Buchli et al. (2006), *f*_4_: force feedback.

The goal of the simulations is to analyse the effects of the different feedback in terms of the stability, transient periods, and power injection that is required from the actuator. Four different instances were taken into account for the sensory feedback dynamics, as illustrated in Figure 2. Although *f*_2_ corresponds to the height of the jump, *f*_4_ is the force that passes through the spring. Then, *f*_1_ and *f*_3_ respectively represent the Tegotae feedback and the feedback that is proposed in Buchli et al. (2006). Interestingly, both of these share a neuro-mechanical coupling. It is evident that all of them introduce a strong polarisation with the critical points, which is defined as *ϕ*_0_. The mechanical parameters and the natural length of the spring are *m* = 0.1 kg, *k* = 5 N/m, *c* = 0.2 Ns/m, and *l*_*0*_ = 1 m, respectively. The parameters of the oscillator are ω = 8 rad/s and *σ* = 2, whose dimensionality is determined on the basis of the feedback law. The initial conditions are respectively *y*_*1*_ = 0.7 m, the velocity is null, and the angle of the oscillator is randomly selected to guarantee a certain robustness with respect to the initial conditions. The actuation parameters and the results of the simulations were obtained from the oscillations in the steady state and are reported in Table 1. The transient period Δ*t* is defined at the point at which the limit cycle is reached. The case *f*_4_ is unable to provide a stable orbit. Finally, it is evident that the introduction of the Tegotae feedback is optimal in terms of the synchronisation transient period. In addition, the energy efficiency *E*_*e*_ is defined by the limit cycle of the period $T^{⋆}$ with the actuation force *F*_*act*_ as follows.

$$E_{e}=\frac{h_{max,T^{⋆}}-h_{min,T^{⋆}}}{E},$$ (28)

$$E=\underset{T^{⋆}}{\int }F_{act}\left(t\right)\dot{h}\left(t\right)dt,$$ (29)

**TABLE 1:** Comparison of performance index, transient period Δ*t*, energy efficiency *E*_*e*_, and power injection *J*, for the feedback types on 1D hopping.

Feedback	*f*_1_	*f*_2_	*f*_3_	*f*_4_
A [N]	4	12	12	4
*ϕ*_0_, Δ [rad]	1.75π0.1π	1.96π0.1π	1.96π0.1π	1.75π0.1π
Δt [s]	3	4	5	∄
*E*_*e*_ [m/Ws]	1.50	1.16	1.15	1.25
J [W]	5.49	17.69	20.15	10.56

Interestingly, to obtain a similar hopping in terms of the height, the cases *f*_2_ and *f*_3_ are required for a higher amplitude of the actuation force.

#### 4.1.2 Robustness and Adaptivity

**FIGURE 3:**
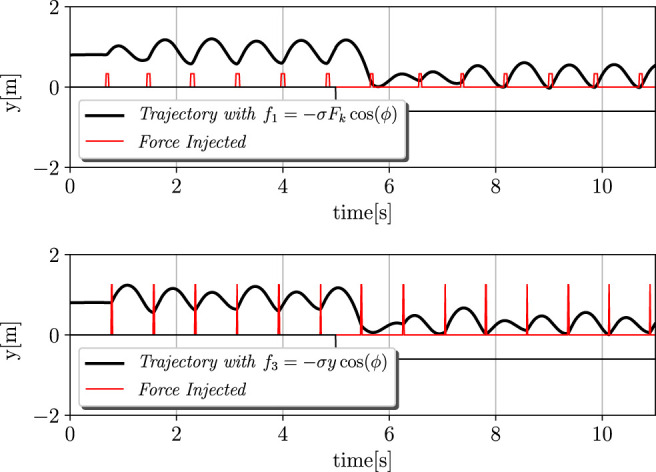
Dynamic environment and adaptation process. The ground level was lowered from 0 to −0.6 m at *t* = 5 s. The upper and lower graphs depict the cases of *f*_1_: Tegotae feedback and *f*_3_: feedback in Buchli et al. (2006). The black and red lines represent the trajectories and force injected, respectively. The Tegotae approach can quickly react to these variations, by modifying the force injection as shown in Eq. 11. The initial state of the monopod robot was the equilibrium point of the spring-mass-damper system. Thus, the height is unchanged while no force is applied.

Second, the case of the Tegotae approach *f*_1_ and the *f*_3_ case that is presented in Buchli et al. (2006) were taken into account. In addition, the adaptivity was evaluated based on the dynamical change in the environment. In particular, at t = 5 s, the ground level was lowered from 0 to −0.6 m. The results are depicted in Figure 3. It is evident that our approach can cope with these variations by performing a proper re-polarisation of the oscillator, even without the adaptation of *σ*, *ϕ*_0_, or Δ. It is possible to notice how the Tegotae approach can quickly react to these variations, by modifying the force injection as shown in Eq. (11).

### 4.2 Case2: Biped

#### 4.2.1 Gait

The objective in the biped case is to first obtain two different gaits, namely in-phase and anti-phase bipedal hopping. As already stated in Owaki et al. (2017); Owaki and Ishiguro (2017b), for the architecture of the CPGs, the frequency of the oscillation *ω* is a useful control variable that can be exploited to introduce a gait transition in the pattern generation. This frequency can be observed as one of the few high-level control variables that are required by CPG architectures, as already presented in Ijspeert (2008). Interestingly, our Tegotae control policy can maintain these properties, even without introducing any oscillator couplings, i.e. $\epsilon_{12}=\epsilon_{21}=0$.

**FIGURE 4:**
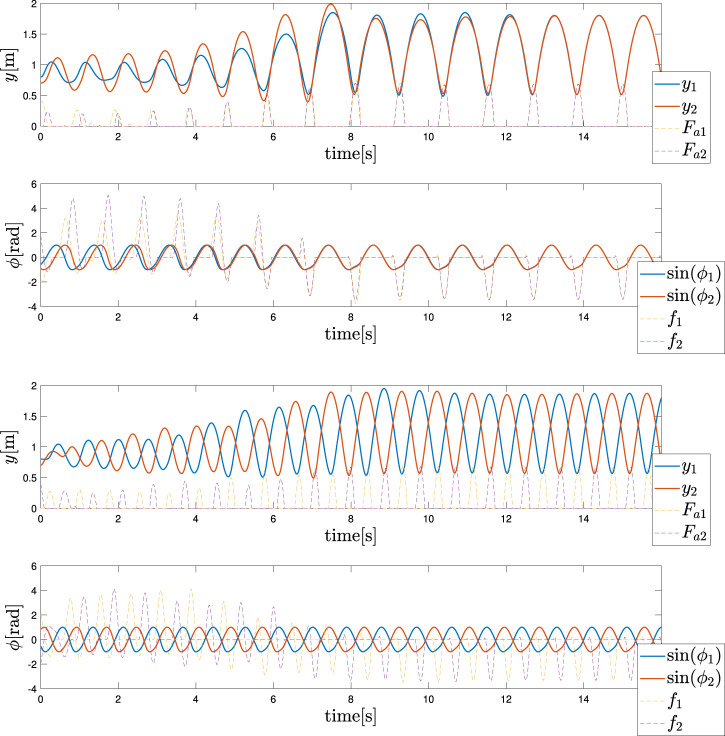
Hopping gait patterns (Top) in-phase hopping: *ω*_*in*_ = 6 rad/s (Bottom) anti-phase hopping: *ω*_*anti*_ = 7.5 rad/s. The upper and lower graphs show the mechanical section (heights and forces) and control section (phases and feedbacks), respectively. The blue and red colors represent the left (1) and right legs (2), respectively.

Two distinct gaits, in-phase hopping and anti-phase hopping, are reported in Figures 4 (Top and Bottom). The case of Figure 4 (Top) is obtained with a frequency *ω*_*in*_ = 6 rad/s, while the second case of Figure 4 (Bottom) is obtained with *ω*_*anti*_ = 7.5 rad/s. At first, we determined these parameters by trial and error. Then, we performed a study on the attractors of the dynamics via Lyapunov Exponents; however, this analysis is out of the scope of this article. The values of the mechanical parameters are generally equal to those in the monoped case, with the addition of a spring constant *k*_*c*_ = 1. The feedback strength was *σ* = 2.4 to guarantee a higher vertical excursion. We considered a few *σ* values, and found that the motion was stable for certain values, while it was unstable for others, suggesting that the value of *σ* has an effect on the stability. However, the effect of *σ* is not considered in this paper because it out of the scope of this study. The initial conditions are *y*_1_ = 0.8 m, *y*_2_ = 0.7 m, the velocities are null, and the angles of the oscillators are selected randomly to guarantee a certain robustness with respect to the initial conditions. These figures represent the mechanical section of the system (heights and forces) and the control section (phases and feedbacks), with the actuation force and Tegotae feedback, respectively.

**FIGURE 5:**
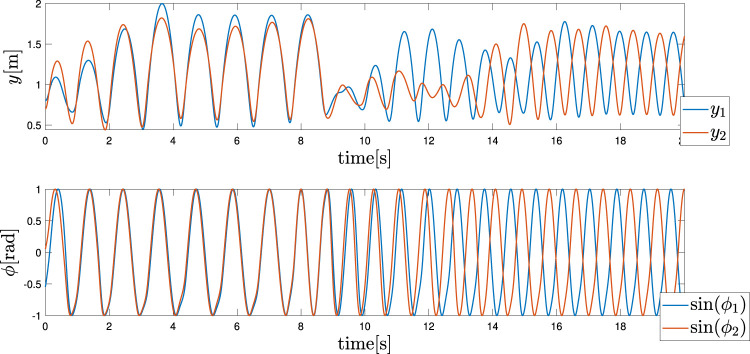
Hopping gait transition. The frequency *ω* is changed from *ω*_*in*_ to *ω*_*anti*_ at *t* = 8 s. The upper and lower graphs depict the height of each leg and phase sin*ϕ*_*i*_ of each leg, respectively.

Finally, it was evident that by changing the control variable from *ω*_*in*_ to *ω*_*anti*_, it is possible to reproduce a gait transition, as depicted in Figure 5. As demonstrated, the value is changed at *t* = 8 s and the trend of the actuation forces and feedback are hidden for clarity reasons due to the presence of several transient sections. The motivations for these specific gaits are shown for the different values of *ω* that are still an open point thus far. This also considers the fact that due to the random initialisation of the phase angles, the other gates are seldomly shown. These cases can be avoided by constructing a more robust architecture that can integrate several types of sensory feedback.

#### 4.2.2 Robustness and Adaptivity

**FIGURE 6:**
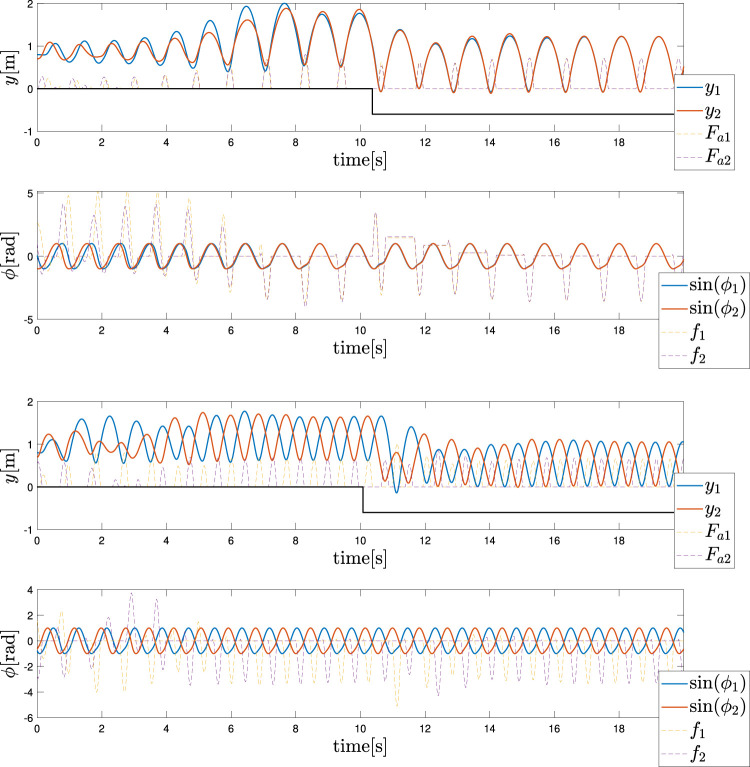
Adaptation to a lower step (Top) In-phase hopping (Bottom) Anti-phase hopping. The ground level was lowered from 0 to −0.6 m at t = 10 s.

Finally, in equivalence to the monoped case, the way in which the control policy expressed in Eq. 11 can sustain a change in the environmental conditions was also examined for the biped case. As depicted in Figure 6A, the ground was first lowered to −0.6 m for both the legs as demonstrated in the monoped case. Meanwhile, the angular frequency was maintained equal to *ω*_*in*_. Second, as depicted in Figure 6B, the ground was lowered again to −0.6 m for both the legs. Meanwhile, the angular frequency was equal to *ω*_*anti*_. The results confirm a good robustness of the control policy to the environmental conditions, which in this case is the ground level.

### 4.3 Optimal Control for the Monoped Case

The optimisation was run for several values of the mass to validate the results for the different feedback dynamics. Meanwhile, all the other parameters were the same as described in the monoped case study. In contrast, the Tegotae controller was applied in Eq. (11) to exploit the adaptivity of the Tegotae feedback.

**FIGURE 7:**
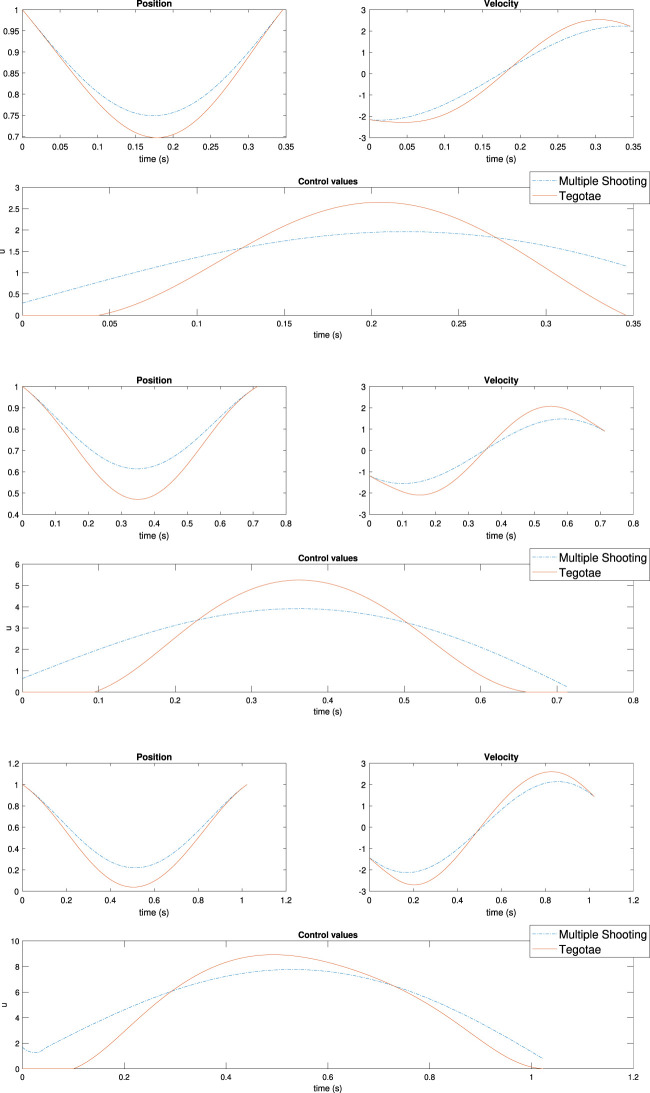
Results of multiple shooting methods. The blue and solid red dotted lines represent the designed optimal controller (MS method) and Tegotae controller, respectively Top case MS1 in Table 2: *m* = 0.1 (Middle) case MS5 in Table 2: *m* = 0.3 Bottom case MS6 in Table 2: *m* = 0.6. Not only was the Tegotae control action extremely similar to the MS optimal control in all the cases, but also the position and velocity profiles demonstrated certain similarities.

The values of the weights for the cost functions are reported in Table 2 with respect to each simulation to determine the effectiveness of the weights. It follows that the actual effect of the weights is restricted to the power injection by the controller. Meanwhile, the optimal controller does not have access to the energy stored in the spring and the damping system or to the vertical excursion, as shown in Supplementary Figures S1–S3 in the Supplementary Material (SM). In contrast, the ability of dynamically adapting to the mass changes of the Tegotae controller is verified by the optimal controller as well, as shown in Figures 7 (Top) to (Bottom). It is evident that the effect of the first term $Q_{2}$ is sufficient to reproduce, for three different values of masses, to reproduce the effects of the Tegotae control. This term corresponds to the energy consumption of the controller. Therefore, the Tegotae control and an optimal control that attempts to maximise the energy efficiency provide similar results for different masses, thereby validating our hypothesis. Further increments of the mass may require a change in the value of *σ* or the use of a non-linear spring to avoid negative values of vertical movements.

**TABLE 2:** Weight values for the cost functions and RMSE *y*, $\dot{y}$, and, *q* for MS.

Simulation	*m*	$Q_{1}$	*R*_1_	*L*_1_	RMSE *y*	RMSE $\dot{y}$	RMSE *q*
MS 1	0.1	1*e*1	−1*e*1	−1*e*1	0.03	0.34	0.58
MS 2	0.1	0	−1*e*1	−1*e*1	0.03	0.34	0.58
MS 3	0.1	1*e*1	0	−1*e*1	0.03	0.34	0.58
MS 4	0.1	1*e*1	−1*e*1	0	0.03	0.34	0.58
MS 5	0.3	1*e*1	0	0	0.03	0.34	0.58
MS 6	0.6	1*e*1	0	0	0.03	0.34	0.58

**FIGURE 8:**
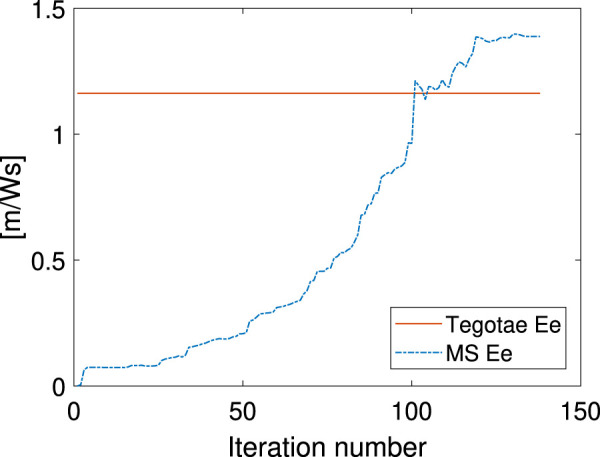
Energy-efficiency convergence in the MS method through comparison with the Tegotae feedback case.

Not only was the Tegotae control action extremely similar to the MS optimal control (see the Supplementary Material) in all the cases, but also the position and velocity profiles demonstrated certain similarities. In all the MS cases, the root mean squared errors (RMSE) were found to be similar, as reported in Table 2, as expected from previous considerations. Finally, for all the cases considered in the MS examples, the energy efficiency of the optimal controller as expressed in Eq. (29) converged to a value similar to that of the Tegotae controller, whose value was determined considering 1 m as the maximum height reached, for comparison purposes. The convergence is reported in Figure 8 for MS1 and leads to a final RMSE of 0.22. This seems to limit to the efficiency given the physical constraints of the system. Moreover, increasing the weight *Q* slightly increases the efficiency.

These results represent the MS case alone. The SS (see the Supplementary Material) has several practical drawbacks, which motivates this choice. First, it requires extremely high weights for the sensitivity function of the final conditions and the smoothness of the control policy. The conditions are automatically satisfied by the continuity constraints in the MS. Second, the convergence is more difficult to obtain. The FHOC for the SS method is formulated by using the norm notation and the additional weights to guarantee a sensitivity to the final conditions and control policy.

$$\underset{q}{\text{min}}\int_{0}^{T}{\left|\left|q_{i}\dot{y}\right|\right|}_{Q_{2}}^{2}+{\left|\left|F_{k}\dot{y}\right|\right|}_{R_{2}}^{2}+{\left|\left|\left(l-y\right)\right|\right|}_{L_{2}}^{2}+{\left|\left|q_{i}\right|\right|}_{S_{2}}^{2}+{\left|\left|q_{i}-q_{i-1}\right|\right|}_{\gamma_{1}}^{2}dt+y_{end}-y{\left(T\right)}_{F_{2}}^{2}+{\left|\left|{\dot{y}}_{end}-\dot{y}\left(T\right)\right|\right|}_{F_{2}}^{2}$$ (30)

Subject to

$$\left[\begin{array}{c} y_{0}-y_{in}\\ {\dot{y}}_{0}-v_{in} \end{array}\right]=0,\left(\text{Initial Value Constraints}\right)$$

$$m\ddot{y}\left(t,q\right)-\left(F_{c}+F_{k}-mg+q\right)=0,\;t\in \left[0,T\right],\left(\text{ODE Constraint}\right)$$

$$\left[\begin{array}{c} y-0-\epsilon \\ -y+l+\epsilon \\ \dot{y}-v_{min}\\ -\dot{y}+v_{max}\\ q_{i}-0-\epsilon \\ -q_{i}+F_{max} \end{array}\right]\geq 0,\;i=0,\ldots ,N,\left(\text{Inequality Constraints}\right)$$

**FIGURE 9:**
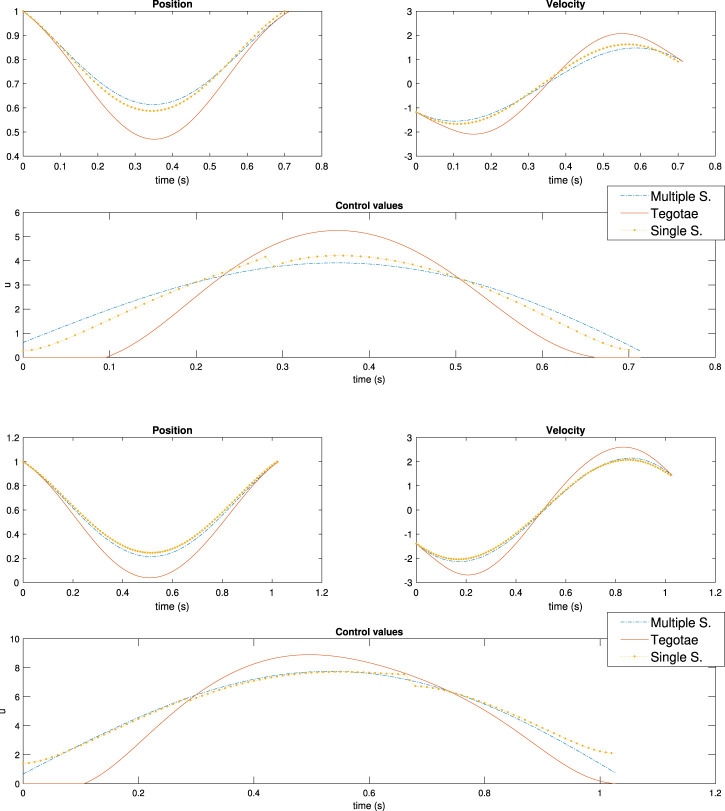
Results of multiple shooting-single shooting (Top) case MS-SS1 in :Table 3 *m* = 0.3 (Bottom) case MS-SS2 in :Table 3 *m* = 0.6.

In our case, *γ*_1_ = 1*e*4 and *F*_2_ = 1*e*10. As previously mentioned, these values are extremely high in comparison with the remaining weights in the cost function as presented in Table 2. Meanwhile, for the MS case, the weights remain the same as MS five in Table 3. Interestingly, it has not been a trivial fact to obtain similar results between the two optimal controllers. It is possible to obtain similar control trends with respect to the MS case, as shown in Figure 9. (Top) and (Bottom) However, there are also cases that are similar to the Tegotae controller, as shown in Supplementary Figures S5,S6 in the SM; this is achieved by varying the values of the weights. For the SS case, the cost function is sensitive to the terms that are proper to the monopod cost function in Eq. 29 and the spring force.

**TABLE 3:** Weights values for the cost functions for the MS-SS.

Simulation	*m*	$Q_{2}$	*R*_2_	*L*_2_	*S*_2_
MS-SS 1	0.3	1*e*1	−1*e*1	−1*e*1	1*e*3
MS-SS 2	0.6	1*e*1	−1*e*1	−1*e*1	1*e*3
MS-SS 3	0.4	1*e*1	−1*e*2	−1*e*2	1*e*3
MS-SS 4	0.6	1*e*1	−1*e*2	−1*e*2	1*e*3

The MS routine is solved by using the interior-point method that is provided by the MATLAB built-in function FMINCON. In contrast, the SS routine is solved by using the BFGS method and the SQP that is designed on the material, provided by Fagiano (2019). With regard to the integration of the dynamics, the time interval was split into 40 nodes with 2 points per sub-interval for the MS case. Meanwhile, a sampling time of 0.01 s was used for the SS case. In both cases, the integration of the dynamics was conducted using an explicit Runge-Kutta method with an order of four since the restricted dynamics were non-stiff. The step size was 0.01 s in both the methods.

## 5 Discussion

The main contribution of this study is to propose a control policy with a reflex-like actuation (Eq. (11)) for the Tegotae-based feedback law in the CPG in such a way that the controller fruitfully exploits the *embodiment* (Pfeifer and Bongard, 2006; Pfeifer et al., 2007). For the validation of the proposed method, we first demonstrated the energy efficiency of the monopod model as well as its robustness and adaptability using the controller. Then, we demonstrated the gait transition for the bipedal model with its robustness and adaptability. Based on the optimal control theory, we designed an optimal controller and then compared it with the Tegotae-based control input. The results indicate the Tegotae-based feedback with reflex-like actuation results for optimal and energy-efficient motion. This suggests the first evidence concerning the optimal energy efficiency for the Tegotae approach.

This study is the first attempt to analyse the optimal energy efficiency along with the adaptivity of the Tegotae approach. Previous studies (Owaki et al., 2017) have mainly focused on the temporal (timing/phase) modulation in the oscillators by the Tegotae feedback on GPG-based models. The proposed reflex-like actuation can modulate the “amplitude” of the actuation via *F*_*a*_ function (Eq. (11)), depending on sensory feedback *F*_*k*_. As presented in Table 1, in comparison with the previous methods, the introduction of the Tegotae feedback *f*_1_ was optimal in terms of the transient period for synchronisation and energy efficiency. The reflex-like pathway (Figure 1A) resulted in a rapid response (fast control loop) on motion generation, leading to the first convergent time in Table 1. Furthermore, the proposed reflex-like actuation (Eq. (11)) induced by the Tegotae feedback in the CPG could generate an input (Figures 7, 9) identical to that of the optimally designed controller, resulting in energy-efficient motion, as presented in Table 1. As discussed in Section. 3, the Tegotae approach has similarities (Eq. (26)) with the *tacit learning* frameworks in Hayashibe and Shimoda (2014). Energy efficiency is also achieved by the accumulation of a quantity that needs to be minimised when directly integrating the state variable. These facts suggest that our control policy, i.e. reflex-like actuation with the Tegotae-based proprioceptive feedback in the CPG, accomplishes optimal energy-efficient motion through the dynamical learning process along with the interaction between the controller, body, and environments (Pfeifer and Bongard, 2006; Pfeifer et al., 2007).

The reflex-based leg coordination models (Ekeberg and Pearson, 2005; Manoonpong et al., 2007; Lewinger and Quinn, 2011; Schilling et al., 2013; Dürr et al., 2019) and reflex-like feedback integration into CPG (Ajallooeian et al., 2013; Dzeladini et al., 2014; Li et al., 2014) have been studied in the past two decades. Pioneering research on “event-driven” reflex models in cats (Ekeberg and Pearson, 2005) and insects (Lewinger and Quinn, 2011; Schilling et al., 2013; Dürr et al., 2019) has been conducted, successfully reproducing various aspects of animal inter- and intra-leg coordination during locomotion. Manoonpong et al. (2007) demonstrated that a reflex-based neural controller could achieve stable and fast bipedal walking. Following the pioneering work integrating a CPG with reflex models (Kimura et al., 1999), similar approaches have been proposed. Ajallooeian et al. (2013); Li et al. (2014) also proposed to integrate a CPG with “event-driven” reflex models for adaptability against perturbations and environmental changes; One of characteristic approaches in this line, Dzeladini et al. (2014) introduced CPG as feed-forward components in reflex-based neuromuscular models for human walking, confirming the idea of using CPGs as feedback predictors (Kuo (2002)) from the viewpoint of gait modulation. In our work, the CPG oscillator is not a feedback predictor, but can be considered as a representation of the movement (phase *ϕ*_*i*_), that is, an internal model. In the Tegotae approcah, the Tegotae function *T*_*i*_(*ϕ*_*i*_,*F*_*k*_) is defined as the product of the function of intended motor command *C*(*ϕ*_*i*_) and sensory information *S*(*F*_*k*_); hence, our reflex-like actuation always modulates the motion based on the Tegotae feedback *f*_*i*_, which increases the value of the Tegotae function *T*_*i*_(*ϕ*_*i*_,*F*_*k*_), leading to its adaptability and optimal energy efficiency, as mentioned in previous paragraph.

Past studies that have used the Tegotae approach (Owaki et al., 2012; Owaki and Ishiguro, 2017b; Owaki et al., 2017) have demonstrated adaptability and behavioural diversity for reproducing animal-like legged locomotion. For quadruped locomotion, the simple and local sensory feedback law in the CPG reproduced the adaptability against the change in mass distribution, which resulted in horse-like or primate-like walking patterns, and a spontaneous gait transition, from walking to trotting and galloping, in response to the locomotion speed. These studies for quadruped robots provide a basis for establishing a design scheme based on the Tegotae approach. For hexapod locomotion, Owaki et al. (2017) designed a minimal model for the inter-limb coordination in a systematic manner based on the Tegotae concept, successfully reproducing the various aspects of the insect locomotion patterns, which includes adaptability to changes in the body properties, e.g. leg amputation. In line with these studies, this investigation also successfully reproduces the adaptability (Figures 3, 6), and behavioural diversity (Figures 4, 5) as well as the energy efficiency. As discussed in previous studies, in the Tegotae approach, the main aim of designing the Tegotae function is to consider the physical consistency of the action and reaction for the desired motion, and to design the Tegotae function such that its value increases in such cases. Once such a Tegotae function is designed, it is possible to modify the control variables in a situation-dependent manner by increasing the value of the Tegotae function as a feedback term ∂T(x,S)/∂x. Therefore, the Tegotae approach enables the design of an autonomous decentralised controller in a systematic manner, by designing the Tegotae function in line with the desired motions.

This study proposes a reflex-like actuation for the Tegotae-based feedback law in the CPG. This is a significant contribution for the actuation and sensory feedback on the adaptation process to the environment and the optimisation process for energy efficiency. However, one of the limitations of this study is that we did not test the applicability of the Tegotae approach to the real-world environment with a physical robot. In addition, it is extremely difficult to perfectly model the dynamics in the real-world environment. One of the key aspects based on the Tegotae approach is the verification in the real world as shown in Owaki et al. (2012); Owaki and Ishiguro (2017b); Owaki et al. (2017). Instead, we analysed the Tegotae control by using the optimal control theory and provided evidence concerning the optimal control input. Regarding the energy efficiency of tacit learning in the real-world environment, it has been verified by achieving a task with a redundant arm in Hayashibe and Shimoda (2018). One potential future direction is to apply our control policy to a robot with more degrees of freedom that performs more complicated tasks. Our control policy is compatible with the force/torque-based control of a physical robot, which is a promising direction of study for future research.

## Data Availability

The original contributions presented in the study are included in the article/Supplementary Material, further inquiries can be directed to the corresponding author.
