# Supplementary material for: Adaptive and Energy-Efficient Optimal Control in CPGs Through Tegotae-Based Feedback
Source: Front Robot AI. 2021 May 26;8:632804. doi: 10.3389/frobt.2021.632804 (PMC8187776; doi:10.3389/frobt.2021.632804)
Supplement: Supplementary file 1 [file DataSheet1.pdf]

Supplementary Material

1 OPTIMAL CONTROL

1.1 Direct Optimal Control Methods

The energy efficiency of the Tegotae approach was analysed by comparing the Tegotae controller with an optimal controller, which was designed by *direct optimal control methods* (Diehl et al. (2005); Bock and Plitt (1984); Fagiano (2019)). The optimization algorithms that are implemented are described in Boyd and Vandenberghe (2009); Nocedal and Wright (2006). *Direct methods* transform the original optimal control problem into a finite dimensional nonlinear programming problem (NLP). This NLP is then solved by the variants of the state-of-the-art numerical optimization methods. Therefore, this approach is often described as "first discretize, then optimize". One of the most important advantages of the direct methods in comparison to the indirect methods is that they can easily treat the inequality constraints. This is because the structural changes in the active constraints during the optimization procedure are treated by well-developed NLP methods that can deal with the inequality constraints and the active set changes. All of the direct methods are based on a finite dimensional parametrization of the control trajectory, but they differ in the way the state trajectory is handled. The direct methods consist of single shooting and multiple shooting. The following section focuses on the latter, since this is a generalization of the first and the algorithm is implemented.

1.2 Single Shoothing Method

The single shooting (SS) method starts by discretizing the controls. We might choose grid points on the unit interval, $0 = \tau_0 < \tau_1 < \cdots < \tau_N = 1$, and then re-scale these grid-points to the possible variable time horizon of the optimal control problem $[0, T]$, by defining $t_i = T\tau_i$ for $i = 0, \ldots, N$. On this grid, the controls $u(t)$ are discretized, so that $u(t)$ only depends on the many finite control parameters $q = (q_0, q_1, \ldots, q_{N-1}, T)$ and it can be denoted by $u(t, q)$. If the problem has a fixed horizon length T, the last component of q disappears since it is not an optimization variable. A numerical simulation routine can be used to solve the initial value problem.

$$\begin{aligned} x(0) &= x_0 \\ \dot{x}(t) &= f(x(t), u(t, q)), \quad t \in [0, T] \end{aligned} \tag{S1}$$

we can now regard the states $x(t)$ in $[0, T]$ as dependent variables. We can denote them by $x(t, q)$. In terms of which simulation routine should be chosen is crucial to the success of any shooting method and it depends on the ODE model type. We also discretized the nonlinear constraints to avoid a semi-infinite problem. For example, by requiring $h(x(t), u(t)) \geq 0$ only at the grid points t_i, a finer grid can also be chosen without any problem. Thus, we can obtain the following finite dimensional NLP.

$$\min_q \int_0^T L(x(t, q), u(t, q))dt + E(x(T, q)) \tag{S2}$$

subject to

$$\begin{aligned} x(0) - x_0 &= 0, && && \text{(Initial Value Constraints)} \\ \dot{x}(t,q) - f(x(t,q), u(t,q)) &= 0, && t \in [0,T], && \text{(ODE Constraint)} \\ h(x(t_i,q), u(t_i,q)) &\geq 0, && i = 0, \ldots, N, && \text{(Non-linear Constraints)} \\ r(x(T,q)) &= 0, && && \text{(Terminal Constraints)} \end{aligned}$$

This problem is solved by a finite dimensional optimization solver, e.g., sequential quadratic programming, which is extensively described in Fagiano2019. The main points of the SS are as follows.

1. It can use fully adaptive, error controlled, state-of-the-art ODEs or DAEs solvers.
2. It only has a few optimization degrees of freedom, even for large ODEs or DAEs systems.
3. Only the initial guesses for the controlled degrees of freedom are needed.

On the other hand, the weak points are described as follows.

1. It is not possible to use the knowledge of the state trajectory x in the initialization (i.e. to track the problems).
2. The ODE solution $x(t,q)$ depends very non-linearly on q.
3. Unstable systems are difficult to treat; in some cases, numerical issues may arise.

This can be partially solved by including a penalty term on the input variations in terms of the cost function, as suggested in Fagiano2019.

1.3 Multiple Shooting

The direct multiple shooting (MS) method tries to combine the advantages of parallel computing with the major advantage of the single shooting (SS) method, namely the possibility to use adaptive, error-controlled ODE solvers. In the MS method, the procedure is as follows. First, the control law is discretized piecewise on a coarse grid as follows.

$$u(t) = q_i \quad \text{for } t \in [t_i, t_{i+1}] \tag{S3}$$

where the intervals can be as large as in the SS. But second, the ODE is solved for each interval $[t_i, t_{i+1}]$ independently, which starts with an artificial initial value s_i.

$$\begin{aligned} x_i(t_i) &= s_i \\ \dot{x}_i(t) &= f(x_i(t), q_i), \quad t \in [t_i, t_{i+1}] \end{aligned} \tag{S4}$$

By numerically simulating these initial value problems, the trajectory pieces $x_i(t, s_i, q_i)$ are obtained. The extra arguments are introduced to denote the dependence on the interval's initial values and controls. Simultaneously with the decoupled ODE solution, the following integral can also be computed as described below.

$$l_i(s_i, q_i) = \int_{t_i}^{t_{i+1}} L(x_i(t_i, s_i, q_i), q_i) dt \tag{S5}$$

In order to constrain the artificial degrees of freedom s_i to physically meaningful values, the following continuity conditions are imposed. $s_{i+1} = x_i(t_{i+1}, s_i, q_i)$. Thus, it has an NLP formulation that is equivalent to the SS NLP, but it contains the extra variables s_i and it has a block sparse structure. $E(s_N)$ is a cost function on the final state s_N, which is different from the one used for the internal states.

$$\min_{s,q} \sum_{i=0}^{N-1} l_i(s_i, q_i) + E(s_N) \tag{S6}$$

subject to

$$\begin{aligned}
s_0 - x_0 &= 0, && && \text{(Initial Value Constraints)}\\
\dot{x_i}(t_{i+1}, s_i, q_i) - f(x_i(t_i, s_i, q_i), q_i) &= 0, && t \in [0, T], && \text{(ODE Constraint)}\\
h(s_i, q_i) &\geq 0, && i = 0, \ldots, N, && \text{(Non-linear Constraints)}\\
r(s_N) &= 0, && && \text{(Terminal Constraints)}\\
s_{i+1} - x_i(t_{i+1}, s_i, q_i) &= 0, && i = 0, \ldots, N-1, && \text{(Continuity Constraints)}
\end{aligned}$$

If we summarize all of the variables as $w \triangleq (s_0, q_0, s_1, q_1, \ldots, s_N)$, we can obtain a NLP in the form of a finite dimensional NLP. In terms of its advantages, the knowledge of the state trajectory can be used in the initialization, and it robustly handles the unstable systems, the path state, and the terminal constraints. In particular, it is interesting to note that the terminal constraint is already satisfied in the first iteration, which is attributed to its linearity. But on the other hand, it needs to be tackled explicitly in the SS, which includes the cost function and some penalty terms for the terminal conditions in order to obtain a sensible trajectory, as suggested in Fagiano (2019). The nonlinear effects of the continuity conditions are distributed over the entire horizon, which is observed in the discontinuities. This is in contrast to the SS, where the non-linearity of the system is accumulated until the end of the horizon. In addition, the terminal constraint becomes more nonlinear than necessary. As stated above, the MS can combine adaptivity with the fixed NLP dimensions, by applying the adaptive ODE/DAE solvers. Within each sequential quadratic programming iteration, the ODE solution is often the most costly part, and it is easy to parallelize. The possibility of using efficient state-of-the-art ODE/DAE solvers and their inbuilt adaptivity makes the MS a competitive option. From a practical point of view, it offers the advantage that the user does not have to decide on the grid of the ODE discretization, but only for the control grid.

1.4 Design of Optimal Controllers

The construction of an optimal controller that is based on the previous methods is non-trivial if a switching system is taken into account. The main issue is passing the information of the switching dynamics and a switching controller to the cost function' s minimizer. As a result, the following scheme has been used. First, the simulation with the Tegotae controller is performed until a steady state is reached. This is done by running the simulation for a sufficiently large time. Second, the peak values of the trajectory for the very last jump are taken. In case these correspond to the flight phase, the dynamics is further cut up into the stance phase for the period only. In fact, these dynamics are the reference dynamics with respect to the optimal controller that needs to be constructed. Afterwards, the flight phase does not need to be tackled by the optimal controller. This is because the control will not be effective in this section. In case if the flight phase is absent, the peak point is directly taken as a reference point. Subsequently, the values of the position and velocity are taken and the finite horizon optimal control (FHOC) problem

is solved by using the MS method. The FHOC problem is solved with respect to these initial and final conditions, which are automatically added by the extended formulation. The cost function is constructed by taking into account Eq. 29 in the main text. The energy stored in the spring system may have a leading role. On the other hand, the maximum height in the vertical excursion is substituted by the length of the body due to the previous considerations about the relevance of the flight phase. The FHOC for the MS method is formulated by using the norm notation $\|x\|_Q^2 = x^T Q x$.

$$\min_{s,q} \sum_{i=0}^{N-1} \left[\|q_i \dot{s}_i\|_{Q_1}^2 + \|(F_k + F_c)\dot{s}_i\|_{R_1}^2 + \|(l - s_i)\|_{L_1}^2 \right] \tag{S7}$$

subject to

$$\begin{bmatrix} s_0 - y_{in} \\ \dot{s}_0 - v_{in} \end{bmatrix} = 0, \qquad \text{(Initial Value Equality Constraints)}$$

$$m\ddot{s}_i(t_{i+1}, s_i, q_i) - (F_c + F_k - mg + q_i) = 0, \quad t \in [0, T], \qquad \text{(ODE Constraint)}$$

$$s_{i+1} - y_i(t_{i+1}, s_i, q_i) = 0, \quad i = 0, \ldots, N-1, \qquad \text{(Continuity Constraints)}$$

$$\begin{bmatrix} s_i - 0 + \epsilon \\ -s_i + l + \epsilon \\ \dot{s}_i - v_{min} \\ -\dot{s}_i + v_{max} \\ q_i - 0 - \epsilon \\ -q_i + F_{max} \end{bmatrix} \geq 0, \quad i = 0, \ldots, N, \qquad \text{(Inequality Constraints)}$$

The second and third terms in Eq. 27 are the energy stored in the spring system and the vertical excursion in the stance phase, respectively. We empirically set these values, Q_1, R_1, L_1 via trial and error.

2 FIGURES

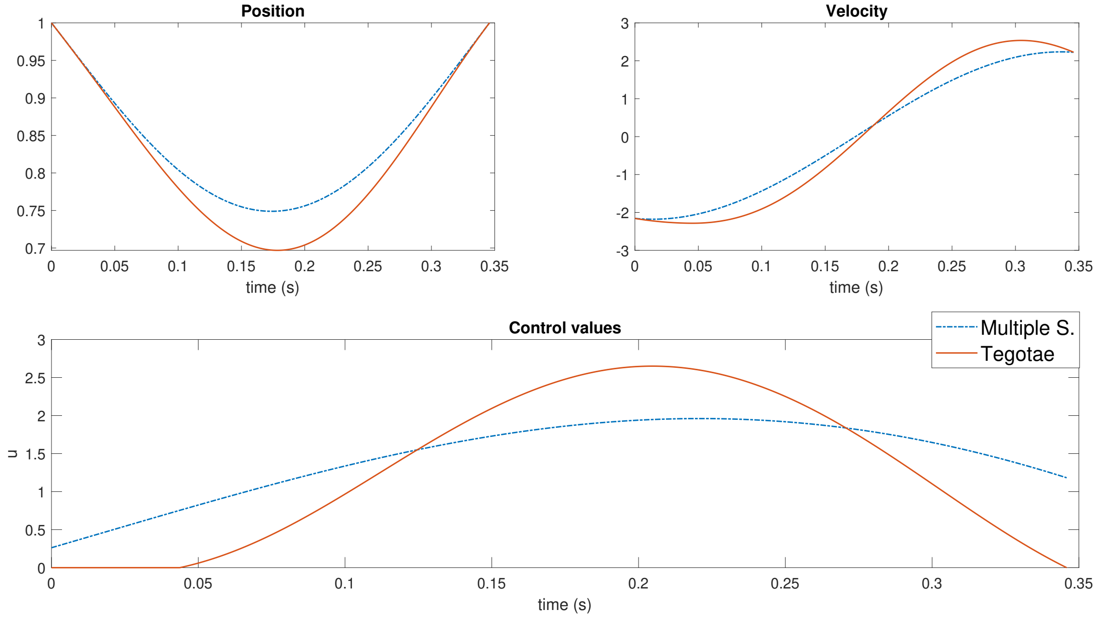

Figure S1. The results of multiple shooting methods. Case MS2 in Table 2: $m = 0.1$. The dotted blue and solid red lines represents the designed optimal controller (MS method) and Tegotae controller, respectively.

REFERENCES

Bock, H. and Plitt, K. (1984). A multiple shooting algorithm for direct solution of optimal control problems. In *Proceeding of IFAC*. 1603–1608

Boyd, S. and Vandenberghe, L. (2009). *Convex Optimization* (Cambdridge University Press)

Diehl, M., Bock, H., Diedam, H., and Wieber, P. (2005). *Fast direct multiple shooting algorithms for optimal robot control* (Fast Motions in Biomechanics and Robotics)

Fagiano, L. (2019). *Constrained numerical optimization for estimation and control* (Lecture notes)

Nocedal, J. and Wright, S. (2006). *Numerical Optimization* (Springer)

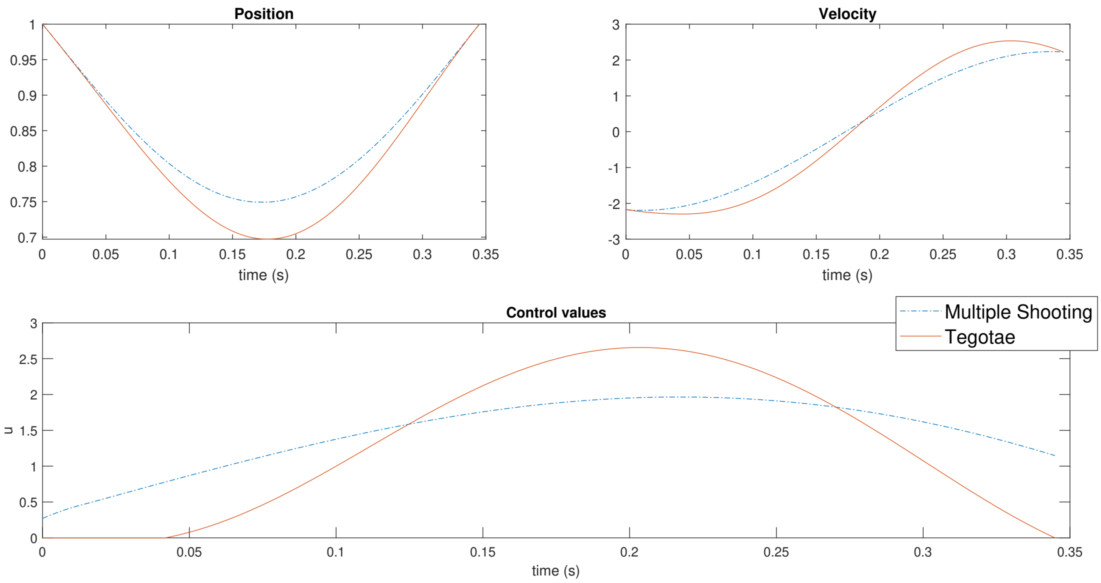

Figure S2. The results of multiple shooting methods. Case MS3 in Table 2: $m = 0.1$. The dotted blue and solid red lines represents the designed optimal controller (MS method) and Tegotae controller, respectively.

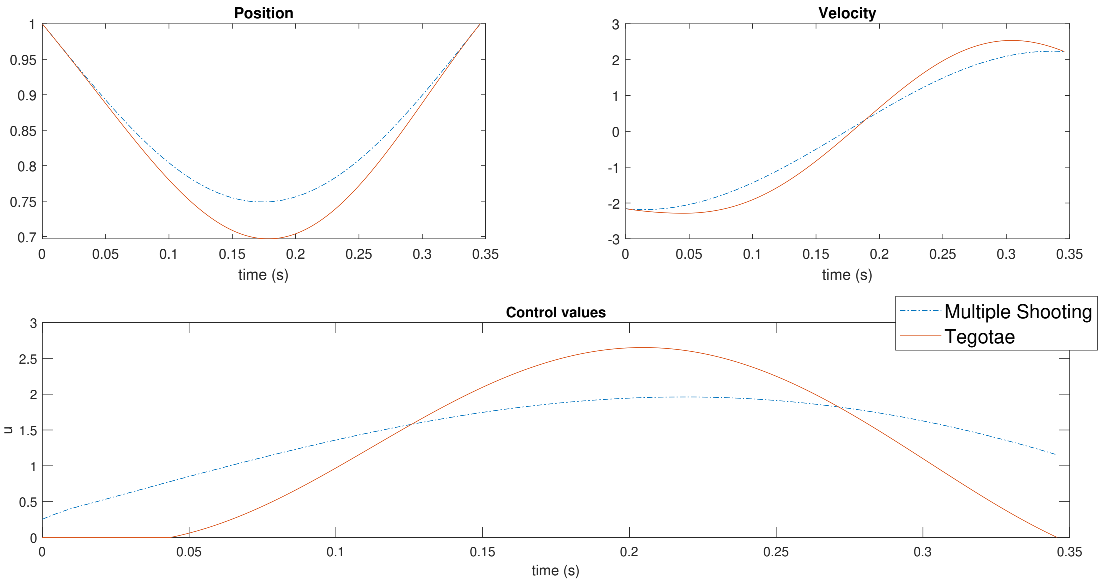

Figure S3. The results of multiple shooting methods. Case MS4 in Table 2: $m = 0.1$. The dotted blue and solid red lines represents the designed optimal controller (MS method) and Tegotae controller, respectively.

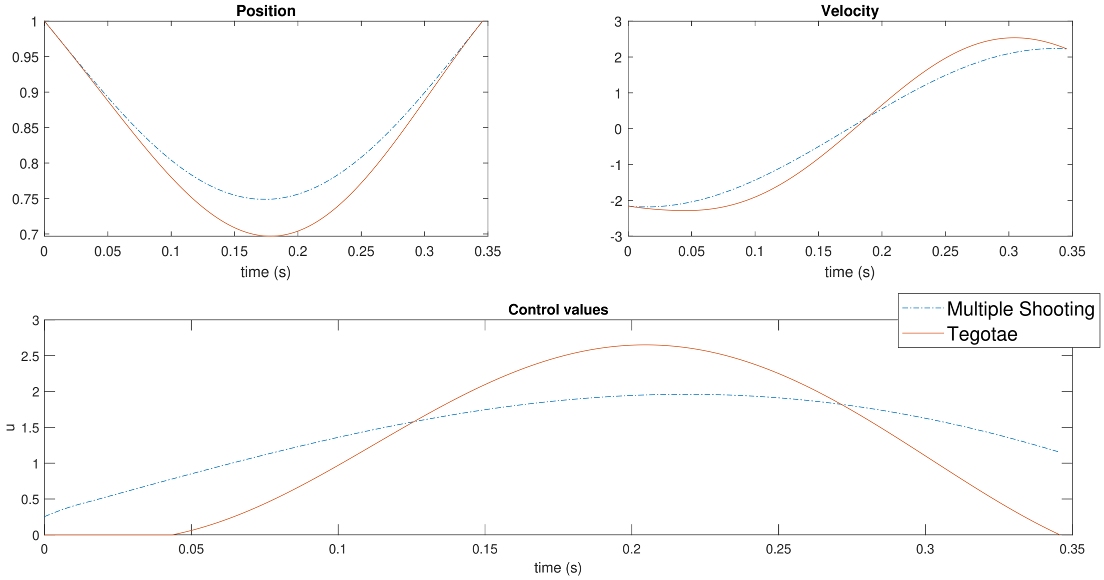

Figure S4. Results of the multiple shooting-single shooting. Case MS-SS3 in Table 3: $m = 0.4$.

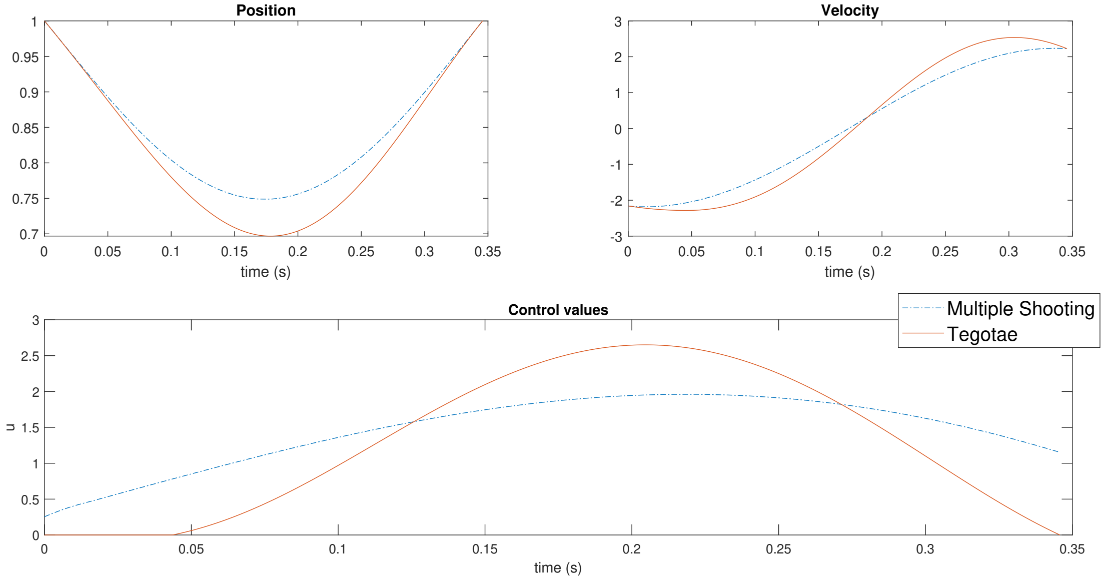

Figure S5. Results of the multiple shooting-single shooting. Case MS-SS4 in Table 3: $m = 0.6$.
